# Systematic Characterization of Degraded Anion Exchange Membranes Retrieved from Vanadium Redox Flow Battery Field Tests

**DOI:** 10.3390/membranes11070469

**Published:** 2021-06-25

**Authors:** Elke Herrmann, Nico Dingenouts, Christina Roth, Frieder Scheiba, Helmut Ehrenberg

**Affiliations:** 1 Institute for Applied Materials (IAM), Karlsruhe Institute of Technology (KIT), 76344 Eggenstein-Leopoldshafen, Germany; frieder.scheiba@kit.edu (F.S.); helmut.ehrenberg@kit.edu (H.E.); 2 Institute for Chemical Technology and Polymer Chemistry (ITCP), Karlsruhe Institute of Technology (KIT), 76131 Karlsruhe, Germany; nico.dingenouts@kit.edu; 3 Faculty of Engineering Science, Electrochemical Process Engineering, University of Bayreuth, 95447 Bayreuth, Germany; christina.roth@uni-bayreuth.de

**Keywords:** vanadium redox flow battery, degradation, anion exchange membrane, field test, real battery system

## Abstract

Commercially available anion exchange membranes were retrieved from VRFB field tests and their degradation due to the various operation conditions is analyzed by in-situ and ex-situ measurements. Ion exchange capacity, permeability and swelling power are used as direct criteria for irreversible changes. Small-angle X-ray scattering (SAXS) and Differential scanning calorimetry (DSC) analyses are used as fingerprint methods and provide information about the morphology and change of the structural properties. A decrease in crystallinity can be detected due to membrane degradation, and, in addition, an indication of reduced polymer chain length is found. While the proton diffusion either increase or decline significantly, the ion exchange capacity and swelling power both are reduced. The observed extent of changes was in good agreement with in-situ measurements in a test cell, where the coulombic and voltage efficiencies are reduced compared to a pristine reference material due to the degradation process.

## 1. Introduction

Vanadium redox flow batteries (VRFBs) are an attractive option for today’s stationary energy storage systems. Multi-MWh VRFB systems have been established for power grid stabilization [1], especially for a growing number of renewable energy applications [2] such as hybrid wind and solar energy systems. According to Skyllas–Kazacos et al. a wide range of systems have already been implemented in Japan, Europe, and the U.S.A. [3,4]. Due to many advantages, such as high storage capacity, quick response, individual scalability of power and capacity (amount of energy), and relatively long cycle life, they show a great potential to improve the reliability and efficiency of electricity distribution networks. However, a full commercialization of VRFB systems is currently limited by several hurdles, such as a relatively low energy density, a poor stability of the utilized electrolyte solutions, high investment costs, and a performance loss due to degradation of battery components [5,6,7]. The cell stack system of the battery involves two half-cells, with redox reactions occurring on commercially available carbon cloth (flow-by) or carbon felt electrodes (flow through). The redox couples V^5+^/V^4+^ in the positive half-cell, and V^3+^/V^2+^ in the negative half-cell, respectively, are dissolved in aqueous sulphuric acid. The exchange membrane physically separates the positive solution (catholyte) and the negative solution (anolyte) in order to prevent self-discharge of the battery [5,8,9]. The long-term stability of individual VRFB components is an imminent point of concern, especially since the cycle stability and long lifetime are the assets that should pay off the high investment costs of VRFBs compared to lithium ion batteries. Therefore, the identification of stressors and an in-depth understanding of the effect and extent of membrane degradation and its influence on the cell performance are essential.

Generally, the demands for the membrane are negligible cross-over (i.e., transport across the membrane) of vanadium ions, low ionic resistance, low cost, and long-term stability in the presence of highly corrosive electrolyte solutions [5,10]. In VRFBs, both anion-exchange membranes (AEM) and cation-exchange membranes (CEM) can be used. The CEM as a proton-conducting membrane can be subdivided into the following four classes: (1) perfluorinated sulfonic acid electrolytes (also PFSA or Nafion^TM^-type), (2) partially fluorinated type, (3) arylene main-chain membrane, and (4) composite CEM [11,12]. Out of these four, PFSA/Nafion membranes show the highest chemical stabilities in VRFBs and are therefore commonly applied in recent studies. Their structure that results from perfluorovinyl ether groups terminated with sulfonate groups onto a tetrafluoroethylene (Teflon) backbone provides them with a specific 3D morphology.

The hydrophobic Teflon back-bone provides the membrane with high mechanical and chemical stability, while the hydrophilic zone, originating from the assembled sulfonated groups, ensures the ion conductivity [9]. Following models were proposed to interpret the morphology of Nafion: The first model for Nafion is called the cluster-network model, also described as ’inverted micelles’ [13]. It consists of an equal distribution of sulfonated ion clusters enclosing a 4 nm diameter pore inside a continuous fluorocarbon lattice. Narrow channels of about 1 nm in diameter interconnect the clusters and form the proton transport channels. Nowadays, the most used model is the water channel or Schmidt–Rohr and Chen model [14]. Many models can give satisfactory descriptions of the scattering pattern. In the Schmidt–Rohr model, also used by Bordin et al. [15] a convincing explanation for the high ion transport capabilities has been given, suggesting oriented cylindrical ionomer channels with quite big diameters (2–4 nm) penetrating the whole membrane. Another important feature of this model is the presence of polymer crystallites in the amorphous phase, not being responsible for the ion transport, but stabilizing the membrane and also the ionomer channels. Small-angle x-ray scattering (SAXS) is an appropriate method to analyze these structural properties. A wide variety of structural models to describe the scattering pattern is reported in literature. As the main structure, the ionomer peak can be described by just three parameters (position, width, and height). Even the origin of the main peak is explained differently, sometimes with an interaction phenomenon (structure factor), but also with shape and contrast in electron density (form factor) [16]. An overview of the structural models can be found in the work of Gebel et al. [17] including the widely used Gierke model [18], one of the first models that explain the ion transport capabilities.

In the VRFB system, besides protons, vanadium ions can enter and cross-over through these water channels. However, the cross-over of differently-charged vanadium species through the membrane between the positive and the negative half-cell causes self-discharge of the VRFB. The transfer of vanadium is accompanied by water transfer (osmotic water and associated water drag), consequently causing a so-called water imbalance which could bring about a chemical shift of the electrolyte. These undesirable processes will significantly reduce the long-term stability and efficiency of the whole system and have to be remedied online by sophisticated balancing strategies [19,20,21]. These technical limitations of CEMs in the VRB system can possibly be alleviated by using anion selective membranes which have a lower permeability for vanadium ions [11,12]. However, AEM generally show the intrinsic disadvantage that the current carriers ${\mathrm{SO}}_{4}^{2-}$(or ${\mathrm{HSO}}_{4}^{-}$) ions inside the AEM matrix exhibit a much lower mobility than exclusively present H^+^ ions in CEMs [22,23,24]. The higher proton mobility in CEMs is possibly attributed to the fact that the proton transport is dominated by the Grotthuss hopping mechanism, leading to much higher conductivities compared to negatively charged species [9].

Some state of the art examples for AEM are polysulfone-based anion exchange membranes, carrying pyridine ion exchange groups (so called Radel membrane) or quaternary ammonium functionalized poly(fluorenyl ether). Hwang et al. [25] described commercial anion exchange polysulfone membranes, cross-linked by a treatment with accelerated electron radiation, which enhanced the cycling performance. Miyabayashi et al. [26] synthesized anion exchange polysulfone membranes, in which polyamine was used as crosslinker. The crosslinking effectively increased the coulombic efficiency (CE), while the voltage efficiency (VE) remained above 85%. Jian et al. [27,28] prepared anion exchanging poly(phthalazinone ether ketone/sulfone) membranes and investigated the effect of the amination agent on these polymers. The ability of the quaternary ammonium group to reduce the vanadium permeability can enhance the ion conductivity. The introduction of new chemical structures, such as blends [29], novel AEMs [30], comb-shaped side chains [31], and block copolymers [32,33] is reported to increase the ion conductivity as well. An industrially produced example for the latter one is FAP450, Fumatech. Its structure has not been disclosed yet in the literature.

In general, it is observed that a higher ion exchange capacity, i.e., a larger amount of fixed ionic groups, will enhance its ion conductivity [34,35,36], while at the same time reducing its chemical stability [37]. The oxidation stability, however, is a major limiting factor in membrane lifetime predictions. This relevant parameter is determined by different methods, such as the Fenton test, extensive cell cycling (fast and up to high potentials), and ex-situ immersion tests in V^5+^-containing electrolyte [9]. The strongly oxidative V^5+^ species in the charged positive electrolyte are considered to mainly be responsible for the so-called membrane fouling process. However, accelerated degradation tests (without any applied potential) might not represent the exact cell operation conditions so that the fouling mechanisms occurring in the cell are not captured precisely. Moreover, it is already well-known from other related fields, e.g., accelerated electrode testing in VRFB [38,39,40,41,42,43,44], that chemical and electrochemical degradation might differ significantly and may also occur on very different time-scales. Since the individual phenomena often cannot be separated, it is difficult to study them in-depth and to develop strategies to mitigate their effects. So far, not much is known about their degradation during operation and to our knowledge, no reports on the systematic analysis of degradation phenomena in AEM retrieved from battery field tests have been published so far.

In this paper, AEMs (FAP450) were retrieved from decommissioned stacks of real battery systems to investigate the authentic ageing of membranes. In our work, the coulombic and voltage efficiencies are investigated in cycling tests so that the degradation state can be identified, while DSC and SAXS analyses give information about the morphology and change of the structural properties. The ion exchange capacity, permeability and swelling power of the membrane are then studied in detail and compared with samples, which were exposed to very different conditions during their “battery life”. This analytical strategy can also be transferred to membranes used in fuel cells or water treatment.

## 2. Materials and Methods

### 2.1. Membranes

In order to analyze authentic membrane fouling, FAP450 anion exchange membranes were retrieved from decommissioned stacks of real battery systems (Schmid, Freudenstadt, D). Six different sheets of membranes were received from three stacks, which remained between one and three years in operation. The first stack was decommissioned after a three-year field test, operated under conservative operation conditions. Subsequently, the membranes of the first and the middle cell were analyzed. In the second battery system, oil contamination of the electrolyte forced a pre-mature shut down. The membranes were taken from the first and the middle cell. The third stack was taken out of operation after a one-year field test under high charging conditions (SOC). The membranes were obtained from the last and the middle cell. The selection of the membranes is on the authority of Schmid, Freudenstadt and shown in Table 1.

As reference membrane (pristine membrane) served a fresh FAP450 anion exchange membrane (Fumatech, Bietigheim–Bissingen, D). The results of the analysed pristine membranes are values with confidential interval. The membranes originate from two batches.

All membranes stated above were soaked in 2.2 M sulphuric acid for at least one day as a conditioning pre-treatment.

### 2.2. In-Situ Cell Test for Determination of the Coulombic Efficiency (CE) and Voltage Efficiency (VE)

The VRB performance is typically determined by its efficiency, which mainly includes coulombic efficiency (*CE*), voltage efficiency (*VE*) and energy efficiency (*EE*). *CE* is the ratio of a cell’s discharge capacity (*Q_dis_*) divided by its charge capacity (*Q_ch_*), described as Equation (1).

(1) $$CE\;\left(\%\right)=\frac{Q_{dis}}{Q_{ch}}\times 100\%$$

The *VE* is calculated via the following equation:

(2) $$VE\;\left(\%\right)=\frac{V_{dis}}{V_{ch}}\times 100\%$$

where *VE* is the voltage efficiency (%), *V_dis_* = discharge voltage and *V_ch_* = charge voltage.

For the *CE* and *VE* determination, a commercially available 10 cm^2^ Micro Flow Cell (Electrocell A/S, Tarm, DK) was used. The cell was equipped with GFD 4.6 carbon felt electrodes (SGL Carbon, Meitingen, D) and a FAP450 anion exchange membrane (Fumatech, Bietigheim–Bissingen, D or Schmid, Freudenstadt, D). Graphite plates served as current collectors. As electrolyte, 70 mL of a commercial 1.6 M vanadium solution in 2 M H_2_SO_4_/0.015 M H_3_PO_4_ (GfE Gesellschaft für Elektrometallurgie mbH, Nürnberg, D) per tank was used.

### 2.3. Ion Exchange Capacity (IEC)

The IEC is related to the amount of ion-exchange groups in the dry ion exchange membrane and indicates the specific interaction between acid and base monovalent ions. For the experimental determination of the IEC, a piece of FAP450 membrane was punched out by a 9 mm diameter punch cutter and conditioned in 2.2 M sulphuric acid for at least one day. The sample was rinsed and placed in 5.0 mL 1.0 M potassium chloride solution for one day. The surface was then washed with deionized water. After drying in air for one week, energy-dispersive X-ray spectroscopy (EDX) (Zeiss Merlin, FE-SEM, 15.0 kV, 300 s, 8000–9000 cps) measurement of the membrane was performed. The chloride proportion in the membrane gave information about the ion exchange capacity (IEC).

### 2.4. Diffusion Measurement Setup and Calculation

The proton diffusion through the membrane is characterized by a specific diffusion coefficient or diffusion constant *D*. In order to determine the constant *D* of protons diffusing through the FAP450 membrane, an in-house built diffusion test setup with two tanks separated by a membrane was used in the experiments. The effective area of the membrane was 4.5 cm^2^. The solutions contained 100 mL of 0.1 M hydrochloric acid solution on the enriched side and 100 mL of 0.1 M of potassium chloride solution on the deficiency side. The decrease of proton concentration in the enrichment side could be neglected. The proton concentration on the deficiency side was measured by a pH meter (Mettler Toledo sevenGo SG2; sensor: Mettler Toledo InLab^®^ 413SG IP67) every minute for 30 min. The experiments were carried out at room temperature.

The diffusion coefficients of protons across FAP450 were calculated using the following equation:

(3) $$ln\left(\frac{c_{A}}{c_{A}-c_{B}}\right)=\frac{DA}{V_{B}L}t$$

in which *D* is the diffusion coefficient of protons (cm^2^ min^−1^); *A* is the effective area of the membrane (cm^2^); *V_B_* the volume of deficiency side (mL); *L* is the thickness of the membrane (cm); *c_A_* is the concentration of protons on the enrichment side (mol L^−1^); *c_B_* is the concentration of protons on the deficiency side (mol L^−1^); and *t* is the test time (min). The volume of deficiency side (*V_B_*) was assumed to be constant, just as the value of *c_A_* by employing a large volume (100 mL) of solution.

When plotting *ln*(*c_A_*/(*c_A_* − *c_B_*)) vs. *t*, the slope which corresponds to the value of (*DA*/*V_B_L*) can be obtained. Consequently, the diffusion coefficient *D* can be calculated from the value of (*DA*/*V_B_L*).

### 2.5. Swelling Ratio (SR) and Membrane Thickness

The dimensional stability was characterized by the swelling ratio (SR) and the determination of the membrane thickness. The swelling ratio was calculated by Equation (4), where the thickness of a dry and a wetted membrane was measured by a micrometer screw (Mitutoyo, 0–25 mm, 0.001 mm).

(4) $$SR\;\left(\%\right)=\frac{\left(Wet\;thickness-Dry\;thickness\right)}{Dry\;thickness}\times 100\%$$

### 2.6. DSC

Differential scanning calorimetry (DSC) allows measurements of the melting and crystallization as well as glass transition temperatures T_g_ of the membranes. Thermal analysis was carried out with a Netzsch DSC 204 (DSC 204 t-sensor E, crucible: DSC-TG pan Al with lid) at a heating rate of 10 K/min under argon atmosphere. Samples were heated from −15 °C to 300 °C. The enthalpies ΔH were calculated by peak integration starting at the onset of the peaks. This method provided information about the changes in the crystalline fraction. The change in the degree of crystallinity of the material ΔX_c_ can be calculated as the ratio between ΔH_c_ and ΔH_0_, where ΔH_c_ is the crystallization enthalpy of the material under study and ΔH_0_ is the crystallization enthalpy of the pristine material (ΔH_0_ = 1267.5 J/g).

### 2.7. SAXS

With Small-angle X-ray scattering (SAXS), changes in the morphology could be observed. Main elements of the scattering behaviour in the angular range observed are the number and size of the crystallites stabilizing the matrix and the size of the transport channels resulting from the self-organization of the sulfonic acid functional groups.

All samples were measured in the following SAXS equipment, using point collimation: A XEUSS 2.0 from Xenocs equipped with a micro focus source and a hybrid photon counting detector (Pilatus 300K-S, Dectris) and a flexible detector distance from 30 cm to 2 m. For the actual measurements, we used 80 cm detector distance, resulting in a range of scattering vector ($\left|\vec{q}\right|=\frac{4\pi \sin\left(\theta \right)}{\lambda }$; 2*θ* is the scattering angle) of 0.08 nm^−1^ to 4 nm^−1^.

The samples were prepared by punching out samples with a 4 mm punch cutter. To receive more scattering intensity, we used 3–4 films together; film thickness was measured with a micrometer screw. Film pieces were stacked in a metal sample holder and closed via adhesive Kapton^®^ foils.

Data handling: Data and background were normalized according to the primary beam intensity measured simultaneously during the measurement. In the next step, the data was corrected first for background scattering, afterwards for sample thickness, and finally for the high-q background resulting from the local density fluctuations on the nm-scale of the amorphous polymer phase.

Modelling of scattering pattern: The scattering pattern of the crystallites was calculated by the use of global scattering functions [45] also called Beaucage-model [46]. It combines a Guinier region and a region of a Porod degree in scattering intensity showing a slope of *q*^−4^ for hard particles or smaller slopes in the case of fractal dimensions.

(5) $$I\left(q\right)=G+B{\left(q^{*}\right)}^{-P}\;\;;\;\;\;q^{*}=\frac{q}{{ [\mathrm{erf}\left(\frac{qR_{G}}{\sqrt{6}}\right)]}^{3}}$$

This description, originally developed for nanoparticles and their aggregates, is used best for particle structures with one homogenous scattering contrast and a high standard deviation in structural size, an assumption valid for crystallites in an amorphous polymer matrix.

## 3. Results and Discussion

The membrane sheets of the AEM were cut into the required number of small pieces for different analyses. Since the particular pieces, however, originate from different positions within the single cell (inlet or outlet, edge or middle), the position may affect the ageing of the membrane, so that the results of the particular analyses cannot be compared directly and may vary slightly. In order to consider such inhomogeneity, multiple analyses were performed, and the results discussed as trends rather than quantitative descriptors.

First of all, the degradation state of the retrieved AEM was determined. Therefore, the electrochemical cycling performance was tested by single cell measurements. Current densities of 25 mA cm^−2^, 50 mA cm^−2^, 62.5 mA cm^−2^, 75 mA cm^−2^, 87.5 mA cm^−2^ and 100 mA cm^−2^ per active area and voltage limits of 0.8–1.6 V were applied.

**Figure 1:**
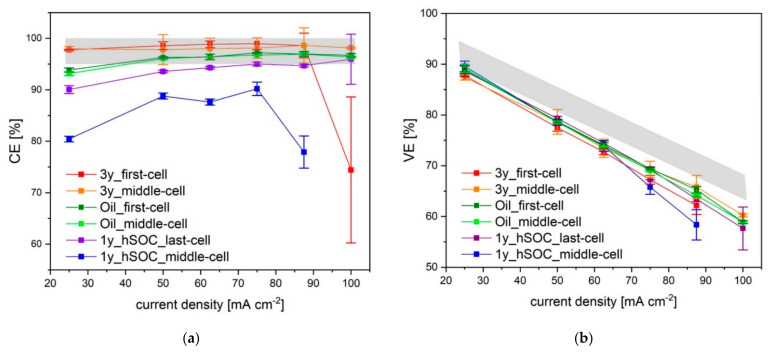
Coulombic efficiencies (**a**) and voltage efficiencies (**b**) of membranes retrieved from decommissioned stacks, grey zone = pristine, standard deviation of five cycles.

Figure 1 shows the resulting coulombic efficiency (CE) and voltage efficiency (VE) of the cycling performance vs. current density. Each experiment was performed for 5 cycles per specific current density. The grey zone in the graph illustrates the approximate confidence interval gained from repetitive results for pristine membranes, which originate from two batches.

In general, the CE appears to increase marginally with an increase in the charging current. This is possibly due to the fact that the charging and discharging process at higher current densities leads to a shorter cycle period. Thus, for the crossover of vanadium species less time is available and therefore charge carrier losses are reduced. Consequently, the CEs increase slightly as the device is cycled.

The membrane aged for one year at a high SOC at the center of the cell stack (1y_hSOC_middle-cell) seems to have the lowest CE-values of all retrieved membranes, which do not exceed 90%. Current densities of 100 mA cm^−2^ cannot be reached in this case. Likewise, the membrane aged for three years at the first cell (3y_first-cell) demonstrates a significant reduction in the cycling performance at high current densities, resulting in a large standard deviation of the CE-values. Specifically, in each discharge cycle a remarkable power-drop effect can be observed that is most probably caused by the decreased conductivity due to blocking of the membrane channels [47]. Membrane 3y_middle-cell exhibits the same effect only in the first cycles. It can be assumed that either the blocking is somehow relieved during extended cycling, or the effect could be caused by the intensity of the current. It is conceivable that high current densities are able to dissolve membrane blocking.

In contrast to 1y_hSOC_middle-cell, membrane 1y_hSOC_last-cell, obtained from the last cell of the identical stack, reveals a CE only slightly below standard. In brief, both came from the same stack, but show significant differences in fatigue. The membrane at the end of the stack was significantly less aged than the one in the middle. This was somehow unexpected in the light of literature [48] and cannot be explained by our measurements.

The oil contaminated membranes Oil_first-cell and Oil_middle-cell (with a maximum of 5 at-% silicones on the surface according to X-ray photoelectron spectroscopy analysis) hardly differ from the pristine membrane.

Consequently, the largest deviation from the pristine membrane is shown by 1y_hSOC_middle-cell and 3y_first-cell, which will be in the focus of our further investigations.

All VEs of the test system decrease with an increase in the charging current due to the increased Ohmic losses and are well below the grey zone, representing test results of the pristine membrane material [49]. Each membrane retrieved from battery field tests shows a lower VE than the pristine membrane. In particular, the VE of 1y_hSOC_middle-cell decreases with increasing current density. A possible reason for this observation can be the increase of the membrane resistance due to loss of ion exchange capacity.

In order to assign this global electrochemical observation to individual processes, the swelling behaviour and ion exchange capacity were analyzed to investigate the decrease in CE, which may originate from changes in permeability and proton diffusivity.

**Figure 2:**
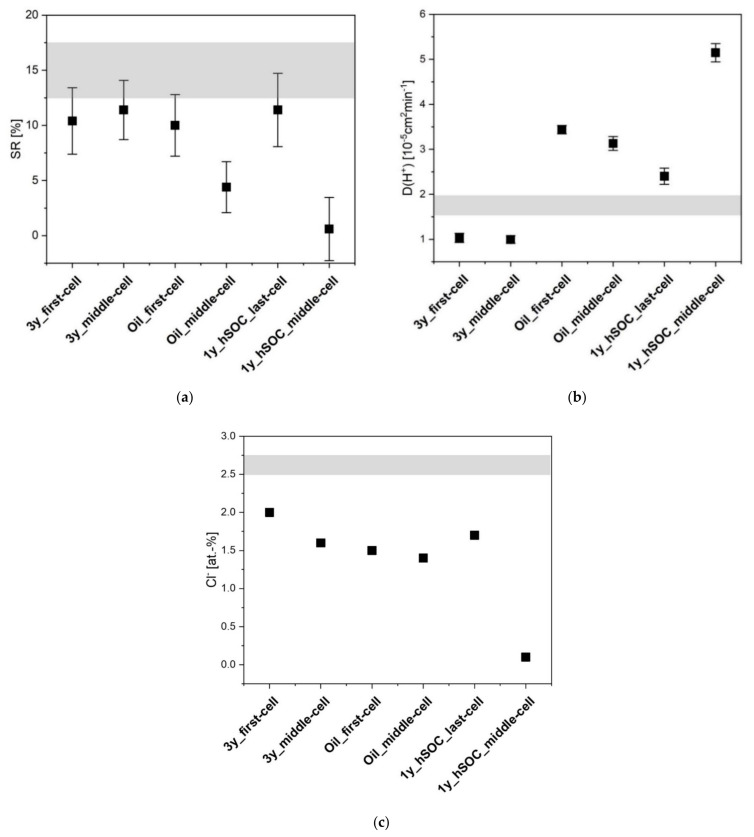
Swelling ratio (SR) (**a**), diffusion coefficients D(H^+^) (**b**) and chlorine ratio of membranes (**c**) retrieved from decommissioned stacks; grey zone = pristine; standard deviation (SD) of swelling ratio includes 10 values and SD of D includes 3 values.

For the analysis of the swelling behaviour, the measurement of the membrane thickness was performed 10 times at various spots for each membrane. The resulting swelling ratios are shown in Figure 2a, where the value including the standard deviation of the pristine membrane is marked in grey. The most significant change in the swelling performance is observed for membrane 1y_hSOC_middle-cell, for which the measured value almost approaches 0%.

The diffusion of ${\mathrm{SO}}_{4}^{2-}$(or ${\mathrm{HSO}}_{4}^{-}$) ions through the membrane is accompanied by osmosis and the permeability of the counter ion H^+^. Latter can be measured and visualized by its diffusion coefficients D(H^+^). The diffusion measurement was performed three times for each membrane. The mean diffusion coefficients and their deviation from the pristine material are depicted in Figure 2b. The values of the AEM from field-tests deviated from the pristine (grey zone) either to lower or to higher values. Extremely high values were observed for membrane 1y_hSOC_middle-cell and extremely low for 3y_first-cell and 3y_middle-cell. Latter findings support the theory of the membrane pore blocking, which is generally referred to a decline in permeability.

A suitable measure for the ion exchange capacity (IEC) is the chlorine amount in the membrane after swelling in potassium chloride solution. To this effect, the membrane was analyzed by energy-dispersive X-ray spectroscopy (EDX). The chlorine ratio of the membrane is shown in Figure 2c. A drop to almost 0% can be seen for the membrane 1y_hSOC_middle-cell.

In brief, membrane 1y_hSOC_middle-cell reveals the most significant deviation from characteristic values of the pristine membrane, showing low CE-values, which do not exceed 90%, almost no swelling behaviour, low IEC, and high permeability. Membrane 3y_first-cell, which could not be tested to high current densities, reveals an extremely low proton diffusion value. The reason for this behaviour might be found in structure variations, which can be probed by DSC and SAXS.

Small-angle x-ray scattering (SAXS) was applied to correlate the materials’ parameters with structural changes in the membrane due to degradation phenomena. According to Bordin et al. [15], who apply the Schmidt-Rohr model, the crystallites are responsible for the main part of low-q scattering, often also called matrix knee. They estimated approximately 3/4 of the intensity resulting from the crystals. Thereby, the low q-part is described more accurately than with models based on lamellar structures.

**Figure 3:**
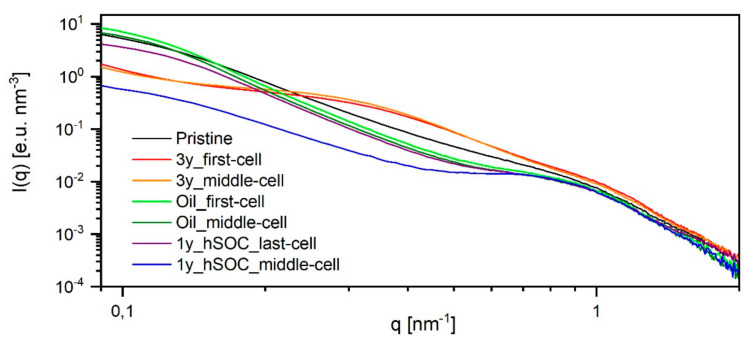
SAXS pattern, scattering data of membranes retrieved from decommissioned stacks and pristine membrane. The data are normalized to primary beam intensity and sample thickness and corrected for background scattering.

The experimental scattering pattern in our work presented in Figure 3 cannot confirm strictly one of the models stated in literature [17] because we measured only in dry state and compared different unknown states of degradation. The scattering patterns show only marginal changes in the ionomer peak region but pronounced changes in the low-q region. Therefore, we decided to base our analysis on the model of Schmidt–Rohr and Chen [14], however, not to perform the full analysis of a two-dimensional structural model followed by a 2D Fourier transform. We just kept the experimentally determined ionomer scattering part constant, interpreting the changes in the scattering pattern by a change in number and average size of the crystallites embedded in the polymer matrix. This analysis allowed us to perfectly describe our data and is in excellent agreement with the results of the DSC-measurements discussed later on.

Figure 3 shows the scattering data of the membranes retrieved from field tests. The ionomer peak is detected slightly below *q* = 1 nm^−1^ for all membranes. This maximum is lower than the one observed in the systems of Bordin et. al. [15] and Schmidt–Rohr and Chen [14]. Therefore, the radius of the water channels determined by a fit with a two-dimensional Beaucage model (radius of gyration of 1.9 nm equals a diameter of 5 nm) is even higher than the range of 2–4 nm found by Schmidt–Rohr and Chen [14].

**Figure 4:**
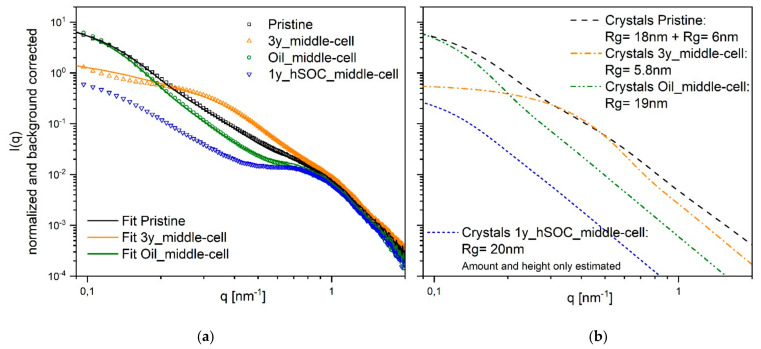
Selected types of SAXS pattern. (**a**): Experimental scattering patterns (points) compared to calculated pattern (solid lines) assuming a constant ionomer scattering part and a changing crystalline part, (**b**): Scattering pattern of the crystalline parts required to explain the difference in the total scattering cures shown in (**a**). For 1y_hSOC_middle-cell the amount of crystallites is only known to be small (from DSC), the average size could be determined. For the other curves, size and amount of crystallites included above the crystallites in 1y_hSOC_middle-cell could be analyzed.

The low q region or the matrix knee differs greatly between the analyzed membranes. We can distinguish four different types of behaviour: Type 1 is that of the original pristine membrane showing an almost continuous degree of scattering intensity. Type 2 (Oil_first-cell, Oil_middle-cell, 1y_hSOC_last-cell) shows a steeper degree and a slightly more pronounced ionomer peak. Type 3 (3y_first-cell, 3y_middle-cell) shows an additional side maximum at around *q* = 0.3 nm^−1^. Finally, type 4 (1y_hSOC_middle-cell) has by far the lowest low-q scattering and the best pronounced ionomer peak. These four different types of scattering patterns are analyzed in the following Figure 4.

As already discussed, we mainly base our analysis on the changes in the crystallites. We can not distinguish if the single crystallites are really changing or if we have aggregates of smaller crystals changing in the number of aggregated crystals. Type 4 (1y_hSOC_middle-cell) has (almost) no crystallites and therefore the lowest low-q scattering. This scattering curve only allowes to analyze the size but not the exact amount of crystallites, resulting in quite large crystallites structures with a radius of 20 nm. To the remaining three types, we could analyze the additional crystalline scattering pattern to explain the difference in the experimental scattering patterns: Type 2 has a similar crystal size compared to type 4, but a much higher proportion of scattering by crystal structures: At low *q*, 86% of the scattering originates from crystals. Type 1, the original membrane, shows a similar amount (79%), but a broader distribution in crystal size requiring smaller crystals in addition. Type 3 shows an extreme drop in the size of the crystalline structures (only 6 nm size and 50% amount). The best way to explain this behaviour could be to assume an original size of the single crystallites of 6 nm (comparable to the assumptions of Schmidt–Rohr and Chen) with different degrees of aggregation in type 1 and type 3. Aggregation to larger structures will increase the scattering intensity without changing the total crystallinity of the polymer. The proportion of scattering originating from crystals to ionomer part is comparable to literature (75% in [14]).

Generally, a structural rearrangement of membrane 3y_first-cell, 3y_middle-cell, and 1y_hSOC_middle-cell can be seen. The patterns for 3y_first-cell and 3y_middle-cell reveal a drop in cluster size and the data for 1y_hSOC_middle-cell indicated a decrease in the number of aggregates.

**Figure 5:**
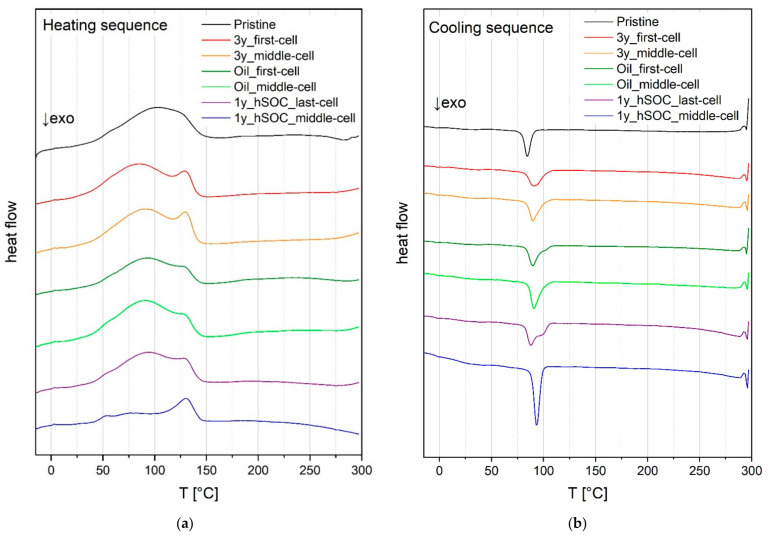
DSC of membranes retrieved from decommissioned stacks and pristine membrane; heating sequence (**a**) and cooling sequence (**b**).

Another identification method for structural changes is the DSC analysis, which is further compared to the SAXS analysis. Figure 5a,b shows endothermic and exothermic processes during melting and crystallization of the membrane material as a function of the temperature. The heating sequence of the pristine membrane reveals one broad peak with a maximum at a melting point (T_melt_) of approx. 100 °C.

For membranes 3y_first-cell and 3y_middle-cell, it can be seen that this broad peak splits into two individual peaks. The first peak has a maximum at 80 °C to 90 °C, the second one a maximum at about 130 °C. Such feature is less pronounced in Oil_first-cell, Oil_middle-cell, and 1y_hSOC_last-cell, while in 1y_hSOC_middle-cell the first peak is hardly present. The splitting of the peaks can be caused by a change in the size of the crystalline clusters. The smaller the clusters, the larger the surface area and the lower the melting temperature of the crystal agglomerate. This behaviour is observed for membrane 3y_first-cell and 3y_middle-cell, where the first peak was 10–20 °C below the meridian T_melt_ of the pristine membrane. The membrane 1y_hSOC_middle-cell reveals only one pronounced peak at T_melt_ = 130 °C. The significantly smaller area of the melting peak (proportional to melting enthalpy Δ*H*_c_) shows a decrease in crystallinity of the polymer. The change in the degree of crystallinity of the material ΔX_c_ is determined as the ratio between Δ*H*_c_ and Δ*H*_0_, where Δ*H*_c_ is the crystallization enthalpy of the material under study and Δ*H*_0_ is the crystallization enthalpy of the pristine material (Δ*H*_0_ = 1267.5 J/g). ΔX_c_ for 3y_first-cell and 3y_middle-cell is 1.1 and therefore similar to the pristine membrane. For membrane 1y_hSOC_middle-cell the value for ΔX_c_ is 0.5, i.e., the crystallinity has halved due to the ageing process. The ΔX_c_ values of the crystallization for all membranes are listed in Table 2.

Comparing the recrystallization peak in the cooling sequence (T_cryst_, in a temperature range from 85 °C to 95 °C), it can be seen that the degree of crystallinity is similar in all samples, also in the membrane 1y_hSOC_middle-cell that has shown a significantly reduced crystallinity before the thermal treatment in the DSC. ΔX_c_, the ratio between Δ*H*_c_ and Δ*H*_0_, shows a value of around 0.1, while Δ*H*_0_ is the crystallization enthalpy of the pristine material in the melting sequence (Δ*H*_0_ = 1267.5 J/g). This demonstrate that the degradation phenomena include reduction in crystal size and frequency, but this can be partially healed up (at least 10%) by melting and recrystallization. The ΔX_c_ values of the recrystallization for all membranes are presented in Table 2.

It can also be observed that all used membranes recrystallize earlier than the pristine membrane; this effect is especially pronounced in the membrane 1y_hSOC_middle-cell. In addition, the recrystallization in this membrane results in the highest crystallinity (highest area of peak with ΔX_c_ of 0.2) of all membranes despite the fact that it was showing the smallest degree of crystallization after usage. An earlier onset of crystallization in a DSC experiment demonstrate reduced requirement of super cooling before the onset of crystallization and indicate a faster crystallization process. This can be explained by a higher mobility in the system, hence, one possible reason might be a degradation of the membranes also in the length of the polymeric chains. Smaller chains show higher mobility and therefore will be able to crystallize faster. Another explanation might be the occurence of additional crystal nuclei due to the degradation process.

To sum up, the degradation can easily be determined in DSC measurements in the remaining crystallinity and in the crystal size distribution detected in the first heating run, but it can also be detected in the recrystallization process after thermal healing.

The comparison of the evaluated SAXS pattern with the DSC analysis shows a structural rearrangement of membrane 3y_first-cell, 3y_middle-cell, and 1y_hSOC_middle-cell for both methods. Moreover, both methods indicate a decrease in crystal cluster size for 3y_first-cell and 3y_middle-cell and a decrease in the number of crystallites in membrane 1y_hSOC_middle-cell, which mean a decrease in crystallinity.

## 4. Conclusions

AEMs from battery field tests with very different operation conditions and from different locations in the stacks were analysed. Membranes that had faced excessive stress by operation in high SOC were compared with ones subjected to only mild operation conditions or oil contamination. Ion exchange capacity, permeability, and swelling power were identified as relevant membrane properties for the resulting cell performance and indicated irreversible changes directly. SAXS and DSC analysis were used as fingerprint methods and provided information about correlated changes in the underlying structure. The most significant deviations from the pristine membrane are observed for the membrane from the center of the stack system, which had undergone a one-year field test under high charging condition (SOC). SAXS and DSC analyses show a decrease in crystallinity and, in addition, an indication of reduced polymer chain length. Two further phenomena are due to the structural changes: First, the water uptake in the membrane decreases compared to the pristine membrane while the diffusion increases. In general, one could have assumed that diffusion is reduced due to the lower water content in the ionomer channels. Second, the IEC is reduced, although the ionomer peak in the SAXS analysis is similar to the pristine membrane. This is because in the SAXS analysis only the structural parameters (e.g., oriented cylindrical ionomer channels) are visible, but not the ion exchange groups. In addition, this method measures the membrane in the dry state, so that water uptake has no influence on the ionomer peak either.

Membranes decommissioned after a three-year field test, either as the first membrane in the stack or from the center of the stack, seem to have reduced permeability, which is supposed to be due to lower proton diffusion induced by blocking molecules. However, the blocking process can be reversible and is not a direct measure for membrane ageing. Its SAXS and DSC analyses shows structural differences between the pristine membrane and the one after three years in use. However, the membrane with high SOC and one-year operation shows the most changes in crystallinity, so that the operating time has a minor influence on the membrane degradation, but excessive stress on the membrane due to high SOC does. Thus, no direct evidence for the influence of operating time on membrane ageing could be obtained so far.

The oil contaminated membranes do not show severe ageing effects. Therefore, membranes with lower than 5 at-% silicones on the surface appear still acceptable for operation.

Membranes after one-year operation with high SOCs from the middle of the stack are more fatigued than membranes from the end of the stack. However, after three-year operation under mild conditions, the first membrane in the stack system is slightly more aged than the middle one. The more persistent blocking processes result in a reduction of CE at high current densities. In fact, the membrane can still be operated, especially when the membrane blocking is reversible.

Differential scanning calorimetry (DSC) is an insightful and simply applicable method for detecting changes in the morphology due to this degradation process and is proposed for the evaluation of accelerated degradation tests.

## Figures and Tables

**Table 1:** FAP 450 membranes, retrieved from decommissioned stacks of real battery systems.

Stack (-Condition)	Membrane	Labeling
3 years	first	3y_first-cell
	middle	3y_middle-cell
Oil contamination	first	Oil_first-cell
	middle	Oil_middle-cell
1 year, high SOC	last	1y_hSOC_last-cell
	middle	1y_hSOC_middle-cell
-	reference	pristine

**Table 2:** FAP 450 membranes, retrieved from field tests and their relative crystallinity’s ΔX_c_.

Membrane	ΔX_c_ Crystallization	ΔX_c_ Recrystallization
3y_first-cell	1.1	0.11
3y_middle-cell	1.1	0.11
Oil_first-cell	0.8	0.11
Oil_middle-cell	1.1	0.13
1y_hSOC_last-cell	1.0	0.14
1y_hSOC_middle-cell	0.5	0.19
pristine	1.0	0.09

## Data Availability

All relevant data generated or analysed during this study are included in this published article. Further data that support the findings of this study are available from the corresponding author on reasonable request but restrictions may apply to the availability of these data due to nondisclosure agreements.
